# Motor Progression in Early-Stage Parkinson's Disease: A Clinical Prediction Model and the Role of Cerebrospinal Fluid Biomarkers

**DOI:** 10.3389/fnagi.2020.627199

**Published:** 2021-01-25

**Authors:** Ling-Yan Ma, Yu Tian, Chang-Rong Pan, Zhong-Lue Chen, Yun Ling, Kang Ren, Jing-Song Li, Tao Feng

**Affiliations:** ^1^Department of Neurology, Center for Movement Disorders, Beijing Tiantan Hospital, Capital Medical University, Beijing, China; ^2^China National Clinical Research Center for Neurological Diseases, Beijing, China; ^3^Engineering Research Center of Electronic Medical Record (EMR) and Intelligent Expert System, College of Biomedical Engineering and Instrument Science, Zhejiang University, Ministry of Education, Hangzhou, China; ^4^Gyenno Science Co. Ltd., Shenzhen, China; ^5^Parkinson's Disease Center, Beijing Institute for Brain Disorders, Beijing, China

**Keywords:** Parksinon's disease, motor progression, predictive model, Parkinson's progression markers initiative, machine learning

## Abstract

**Background:** The substantial heterogeneity of clinical symptoms and lack of reliable progression markers in Parkinson's disease (PD) present a major challenge in predicting accurate progression and prognoses. Increasing evidence indicates that each component of the neurovascular unit (NVU) and blood-brain barrier (BBB) disruption may take part in many neurodegenerative diseases. Since some portions of CSF are eliminated along the neurovascular unit and across the BBB, disturbing the pathways may result in changes of these substances.

**Methods:** Four hundred seventy-four participants from the Parkinson's Progression Markers Initiative (PPMI) study (NCT01141023) were included in the study. Thirty-six initial features, including general information, brief clinical characteristics and the current year's classical scale scores, were used to build five regression models to predict PD motor progression represented by the coming year's Unified Parkinson's Disease Rating Scale (MDS-UPDRS) Part III score after redundancy removal and recursive feature elimination (RFE)-based feature selection. Then, a threshold range was added to the predicted value for more convenient model application. Finally, we evaluated the CSF and blood biomarkers' influence on the disease progression model.

**Results:** Eight hundred forty-nine cases were included in the study. The adjusted *R*^2^ values of three different categories of regression model, linear, Bayesian and ensemble, all reached 0.75. Models of the same category shared similar feature combinations. The common features selected among the categories were the MDS-UPDRS Part III score, Montreal Cognitive Assessment (MOCA) and Rapid Eye Movement Sleep Behavior Disorder Questionnaire (RBDSQ) score. It can be seen more intuitively that the model can achieve certain prediction effect through threshold range. Biomarkers had no significant impact on the progression model within the data in the study.

**Conclusions:** By using machine learning and routinely gathered assessments from the current year, we developed multiple dynamic models to predict the following year's motor progression in the early stage of PD. These methods will allow clinicians to tailor medical management to the individual and identify at-risk patients for future clinical trials examining disease-modifying therapies.

## Introduction

Parkinson's disease (PD) is a chronic progressive neurodegenerative disorder characterized by a broad spectrum of gradual motor and non-motor impairments (Selikhova et al., 2009). In the clinical course of PD, both linear (Gottipati et al., 2017; Holden et al., 2018) and non-linear (Vu et al., 2012; Reinoso et al., 2015) progression have been reported in the advancement of motor and non-motor symptoms. In an era of increasing focus on individualized management and disease-modifying therapies, there is a need to develop tools to predict motor progression at the individual level. However, the substantial heterogeneity (Foltynie et al., 2002; Selikhova et al., 2009; Ma et al., 2015; Qian and Huang, 2019) of clinical symptoms and lack of reliable progression markers present a major challenge in predicting accurate progression and prognoses.

The current literature on PD progression consists largely of associative analyses focusing on predictors such as gender, age, clinical subtype (Aleksovski et al., 2018), genes (Deng et al., 2019), cognitive status and baseline motor score (Reinoso et al., 2015). Greater progression of motor scores has been associated with several factors, such as male gender, older age at diagnosis, akinetic-rigid subtype, cognitive impairment (Reinoso et al., 2015), right-side onset (Baumann et al., 2014), orthostatic hypotension, and rapid eye movement sleep behavior disorder (Fereshtehnejad et al., 2015). Apart from associative analyses, a few prognostic models have been developed that focus on predicting different aspects of PD at the individual level, including logistic regression and Bayesian classification models to predict cognitive impairment (Schrag et al., 2017; Hogue et al., 2018; Gramotnev et al., 2019), machine-learning, random survival forests to predict time to initiation of symptomatic treatment (Simuni et al., 2016) and Bayesian machine-learning methods to predict motor progression (Latourelle et al., 2017) and data mining and classification techniques to predicting faster symptoms worsening at baseline patients evaluation (Tsiouris et al., 2017). Based on the Parkinson's Progression Markers Initiative (PPMI) database, Latourelle et al. developed comprehensive multivariable prognostic models to predict the annual rate of change in PD (Latourelle et al., 2017). The authors included relatively comprehensive indicators, including baseline molecular and clinical variables, to construct an ensemble of models to better clarify and analyze the predictors. However, due to the large heterogeneity of motor symptoms and the complexity of related factors, it was difficult to predict motor progression with high accuracy. The models yielded a cross-validated *R*^2^ value of 41% for the PPMI cohort and 9% for the LABS-PD cohort.

Increasing evidence indicates that each component of the neurovascular unit and blood-brain barrier (BBB) disruption may take part in many neurodegenerative diseases (Yamazaki and Kanekiyo, 2017). Since some portions of Cerebrospinal Fluid (CSF) are eliminated along the neurovascular unit and across the BBB, disturbing the pathways may result in changes of these substances. CSF biomarkers in PD, such as α-synuclein species, lysosomal enzymes, markers of amyloid and tau pathology, and neurofilament light chain, have been suggested to possess potential diagnostic and prognostic value of PD (Parnetti et al., 2019). In large areas of undeveloped countries, costly tests, such as genetic and CSF testing, and image detection for PD (PET and MIBG), could exert a great burden on patients and medical security systems. Furthermore, the corresponding variables are not data routinely used for common clinical activities and are difficult to obtain. Considering clinical needs and utility, by using machine-learning methods with PD patient data from the PPMI database, we aim to develop multiple dynamic models to predict motor progression based on general information and classical clinical scales, displayed in the form of the Movement Disorder Society-Unified Parkinson's Disease Rating Scale (MDS-UPDRS) Part III score. We also explore the influence of CSF and blood biomarkers on the prediction model. The use of baseline assessments (e.g., age, gender, disease duration, motor and non-motor examination, validated self-report questionnaires), which are either already routinely performed or could be reasonably implemented during a typical neurologist's office visit, can facilitate widespread implementation of this cost-efficient predictive model in real world applications.

## Methods

### Participants

Data used in the preparation of the article were obtained from the PPMI database. The PPMI is an international, multicenter, prospective study designed to discover, and validate biomarkers of disease progression in newly diagnosed PD participants (National Clinical Trials identifier NCT01141023). Each PPMI recruitment site received approval from an institutional review board or ethics committee on human experimentation before study initiation. Written informed consent for research was obtained from all individuals participating in the study. The PPMI database was accessed on December 9, 2020, to obtain data from baseline visits (*n* = 474) and follow-up visits (the numbers of follow-up visits for five separate years are 380, 323, 257, 259, 230). For up-to-date information on the study, please visit www.ppmi-info.org.

### Model Variables

The main model outcomes of interest were motor function in the coming year. The primary outcome was the MDS-UPDRS Part III score. Predictive variables included demographics, disease duration, and measures of motor (off state) and non-motor function. Motor assessments included the MDS-UPDRS Parts II-IV, Hoehn and Yahr Stage (H&Y), PD subtype evaluations (tremor dominant (TD), postural instability and gait difficulty (PIGD) and TD/PIGD scores). For non-motor evaluation, the Epworth Sleepiness Scale Score (ESS) and REM Sleep Behavior Disorder Screening Questionnaire Score (RBDSQ) were applied to measure a patient's sleep quality and disturbances. The Questionnaire for Impulsive-Compulsive Disorders in Parkinson's Disease (QUIP) score was used to evaluate PD impulse control disorders. The Geriatric Depression Scale (GDS) score was designed specifically to screen for depression in geriatric patients. The total score of the assessment of autonomic dysfunction in Parkinson's disease (SCOPA-AUT) was used to evaluate autonomic nervous function in the prior month. The MDS-UPDRS Part I score and component scores were used to evaluate mood and mentation.

In addition, we assessed whether biomarkers including CSF amyloid, CSF α-synuclein (and its ratio to CSF amyloid), serum uric acid had an impact on the progression model in the study.

### Data Preprocessing

Each patient had a maximum of five samplings performed at different intervals. During data processing, we excluded negative differences in the MDS-UPDRS Part III score between the coming year and the prior year. According to the PPMI protocol, the “off state” was evaluated as more than 6 h after the last dose of dopaminergic therapy. Considering that the disease was always in a progressive state for all patients, a decrease in the MDS-UPDRS Part III score with increasing disease duration was thought to be unrealistic; therefore, patient samples that displayed this trend were discarded. Furthermore, as eliminating redundant information can improve the quality of the prediction model, we performed Spearman rank correlation analysis to ensure that no variables were correlated with a correlation coefficient of more than 0.5. Prior to model construction, all variables were transformed to a z-score by subtracting the mean value from each of the observed values and dividing by the standard deviation because the clinical phenotypes were measured on different scales and with different score ranges.

### Regression Model Construction

First, the preprocessed data were divided into training and test sets. There were two dividing principles: (1) After ordering by diagnosis date, the first 75% of patients were included in the training data set; the remaining patients were included in the test set. (2) To avoid data overlapping, samples from the same patient were placed into either the training or test set.

We used five regression algorithms, including linear, ridge, Bayesian, random forest, and gradient boosting decision tree regression, to build the model with the training set. Features were selected using the recursive feature elimination (RFE) method. By evaluating model performance, the most irrelevant variables were eliminated in each iteration of the regression, and then the contributions of the remaining variables to the model were ranked. To select the best combination of features, RFE was performed with cross-validation to calculate the verification error of all subsets of features. The subset with the smallest error was regarded as the final feature combination. Next, regression models were built with parameter adjustment to achieve the best performance. Finally, the models were validated with the test set.

### Assessment of Biomarkers' Influence on Parkinson's Disease Progression Model

Due to the great difference between the absence of biomarkers and other clinical variables, the initial inclusion would result in smaller amounts of data. So, biomarkers were added after model construction based on the clinical and scale scores data to evaluate their influence on the basic model. First, correlation analysis was performed between biomarkers and disease progression. Then the variables with significant correlation were selected to improve the models and we compared the model difference between before and after adding the biomarkers variables.

### Statistical Analysis and Performance Measures

Descriptive statistics of the patients' demographic and clinical characteristics are summarized in Table 1. The Kolmogorov-Smirnov test was used to test the normality of continuous data. Continuous variables are described by the mean, standard deviation, maximum, and minimum. Categorical variables are expressed as percentages. The *t*-test was used to compare regression models before and after adding biomarkers variables. Two-tailed *p* < 0.05 were considered to indicate statistical significance.

**Table 1:** Descriptive statistics of the demographic and clinical variables of the Parkinson's progression markers initiative study participants.

**Variables**	**Mean (SD; Min; Max)**	**Percentage (%)**
Age	61.473 (9.753; 33.498; 84.884)	
Gender		Male: 65.823
		Female: 34.177
Age at symptom onset	59.460 (10.054; 25.370; 83.008)	
Side most affected at PD onset		Left: 39.873
		Right: 56.962
		Symmetric: 3.165
Family History of PD		First degree family w/PD: 14.768
		Non-1st degree family w/PD: 11.392
		No family w/PD: 73.840
Duration (Months)	6.710 (6.664; 0.400; 37.000)	
Total rigidity score	3.508 (2.703; 0.000; 13.000)	
Hoehn & Yahr stage		Stage 1:45.992
		Stage 2:53.376
		Stage 3: 0.633
TD/PIGD classification (OFF)		TD: 71.730
		PIGD: 17.300
		Indeterminate: 10.970
Tremor score (OFF)	4.340 (3.105; 0.000; 18.000)	
MDS-UPDRS part III score (OFF)	19.937 (9.141; 2.000; 51.000)	
ESS score	6.162 (3.758; 0.000; 20.000)	
RBDSQ score	4.162 (2.686; 0.000; 12.000)	
MOCA score	27.133 (2.325; 17.000; 30.000)	
QUIP score	0.325 (0.697; 0.000; 4.000)	
GDS score	2.464 (2.672; 0.000; 14.000)	
SCOPA-AUT total score	10.103 (6.760; 0.000; 44.000)	
MDS-UPDRS part I score	5.956 (4.571; 0.000; 27.000)	
Initial symptom (at diagnosis)—resting tremor		Symptom present at diagnosis: 78.692
Initial symptom (at diagnosis)—rigidity		Symptom present at diagnosis: 73.840
Initial symptom (at diagnosis)—bradykinesia		Symptom present at diagnosis: 82.068
Initial symptom (at diagnosis)—postural instability		Symptom present at diagnosis: 7.595

*SD, Standard deviation; Min, Minimum; Max, Maximum; TD, Tremor Dominant; PIGD, Postural Instability/Gait Difficulty; MDS-UPDRS, Movement Disorder Society-Unified Parkinson's Disease Rating Scale; ESS, Epworth Sleepiness Scale; RBDSQ, REM Sleep Behavior Disorder Screening Questionnaire; MOCA, Montreal Cognitive Assessment; QUIP, Questionnaire for Impulsive-Compulsive Disorders in Parkinson's Disease; GDS, Geriatric Depression Scale; SCOPA-AUT, Assessment of autonomic dysfunction in Parkinson's disease*.

The performance of the models was assessed by the root mean square error (RMSE) and the adjusted coefficient of determination (adjusted R-squared coefficient or adjusted *R*^2^). *R*^2^ is a statistical measure in the regression model, equal to the ratio of the regression sum of squares to the total sum of squares and which reflects the degree of agreement between the data and the model. The influence of the number of variables on the goodness is excluded in the adjusted *R*^2^. Because doctors may score the same patient with minor differences in terms of the clinical significance of the symptom, we added a threshold range to each predicted value, which acted as the midpoint of the range, observed whether the true value fell within the range and calculated the accuracy for more convenient application of the model.

## Results

### Patient Demographic and Clinical Characteristics

**Figure 1:**
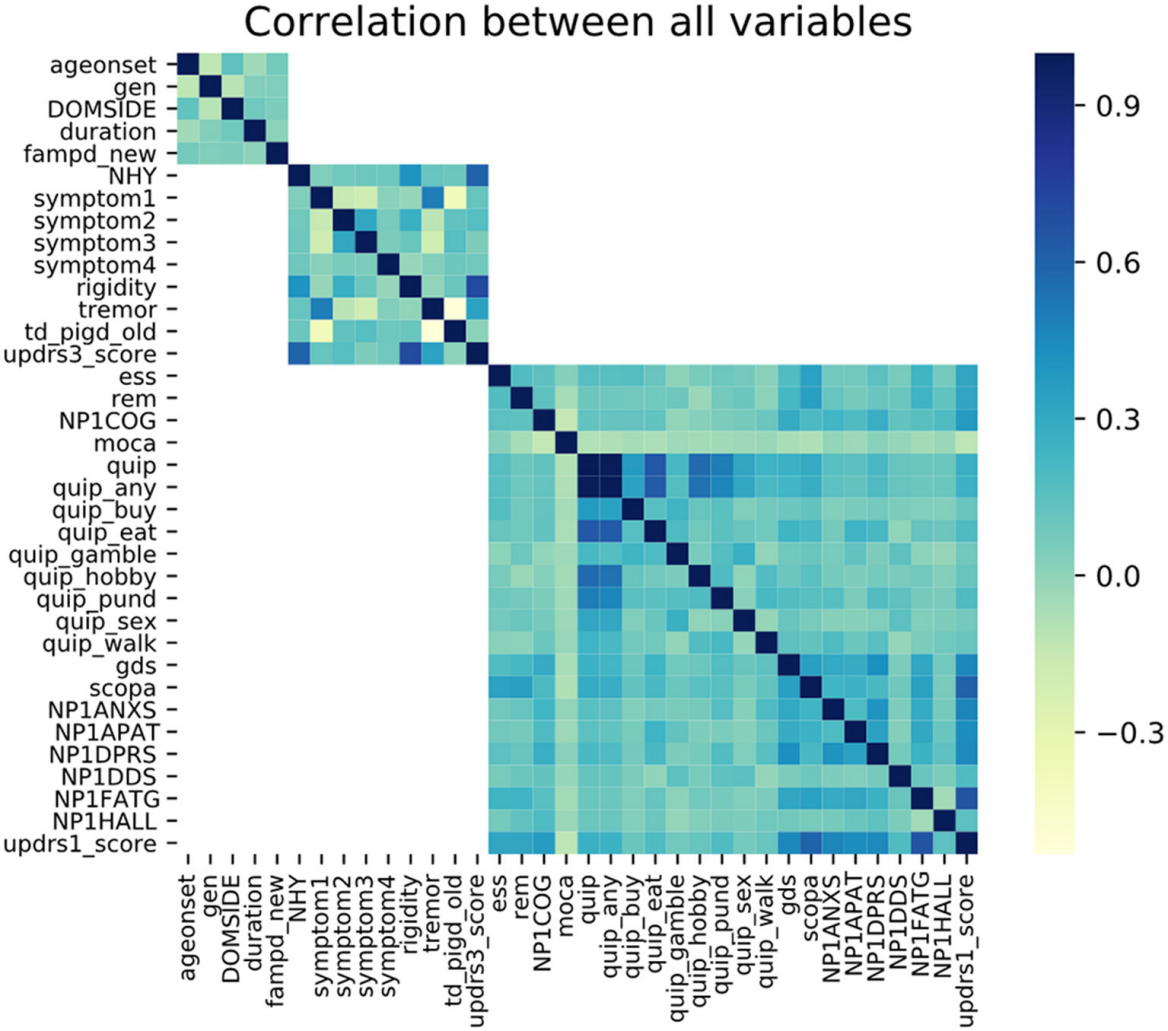
Correlation heatmap between all variables.

In this study, 474 (312 males and 162 females; M:F ratio 1.926:1; mean age 61.473 ± 9.753 years) PD patients were enrolled. The mean age of disease onset was 59.460 years, and the mean disease duration was 6.710 months at baseline. None of the continuous data were normally distributed according to the Kolmogorov–Smirnov test. Descriptive statistics of the general and total score variables are shown in Table 1 and other detailed score variables are shown in Supplementary Table 1. A heatmap of the Spearman correlation coefficients between the variables are depicted in Figure 1. To reduce redundant information, we eliminated variables with strong correlations. So, most of the individual items were removed and the total scores were retained. In total, 24 features remained and are shown in Table 2. Apart from these, we also calculated the correlations between the 24 individual variables and the outcome event. The variables with the top five highest correlations were MDS-UPDRS Part III Score (0.882), Duration of Disease since Diagnosis (0.269), MDS-UPDRS Part I Apathy (0.197), SCOPA-AUT Total Score (0.196), and MDS-UPDRS Part I Fatigue (0.154). The correlation coefficients and probability values of all 24 variables are shown in Supplementary Table 2.

**Table 2:** The remaining 24 features after eliminating features with strong correlation.

**Category**	**Features**
General	Age at symptom onset gender side most affected at PD onset Duration (Months) family history of PD
Motor	Four initial symptoms at diagnosis TD/PIGD classification MDS-UPDRS Part III Score
Non-motor	ESS score RBDSQ score QUIP score GDS score SCOPA-AUT total score MOCA score MDS-UPDRS part I analytic score

### Prediction Model Construction and Performance Measures

After eliminating the missing data, a total of 849 cases were left, among which 633 cases were placed in the training set and 216 in the test set according to the aforementioned dividing principles. The disease progression as expressed by the MDS-UPDRS Part III score of the coming year was predicted based on the information from the previous year. We built regression models based on five algorithms, and their performances are shown in Table 3. The models can be divided into three categories: linear, Bayesian and ensemble methods. The adjusted *R*^2^ of all the models reached above 0.75. The feature combinations obtained by the RFE method for models in the same category were similar and the feature importance or coefficient of each model is shown in Table 4. The models from each of the categories jointly selected the same three features: MDS-UPDRS Part III score, MOCA Score and RBDSQ score. All coefficients of regression models are shown in Table 5.

**Table 3:** The performance of five regression models.

**Model category**	**Model**	**Adjusted *R^**2**^***	**RMSE**
Linear methods	Linear regression	0.779	5.585
	Ridge regression	0.779	5.584
Bayesian method	Bayesian regression	0.778	5.579
Ensemble methods	Random forest	0.770	5.678
	Gradient boosting	0.753	5.918

**Table 4:** The feature importance or coefficient of features selected through RFE method in five regression models.

**Features selected through RFE method**	**Feature importance or coefficient**				
	**Linear regression**	**Ridge regression**	**Bayesian regression**	**Random forest**	**Gradient boosting**
MDS-UPDRS part III score (OFF)	0.970	0.970	0.976	0.929	0.958
Age at symptom onset	/	/	/	0.009	0.002
Duration (months)	/	/	/	0.028	0.030
Family history of PD	0.523	0.521	0.326	/	/
MDS-UPDRS part I anxious mood	0.484	0.479	0.209	/	/
RBDSQ score	0.151	0.151	0.103	0.010	0.007
MDS-UPDRS part I features of dopamine dysregulation syndrome	−1.780	−1.738	−1.302	/	/
Initial symptom (at diagnosis)—postural instability	/	/	0.427	/	/
ESS score	/	/	/	0.010	/
SCOPA-AUT total score	/	/	/	0.007	/
MOCA score	−0.031	−0.031	−0.001	0.007	0.003

**Table 5:** All coefficients of regression models.

**Model**	**Coefficients**
Linear regression	/
Ridge regression	Regularization strength = 1.32
Bayesian regression	Maximum number of iterations = 300
Random forest	The number of trees in the forest = 260
	The maximum depth of the tree = 4
	The function to measure the quality of a split = “the mean absolute error”
Gradient boosting	The number of boosting stages to perform = 30
	Learning rate = 0.1
	The function to measure the quality of a split = “the mean squared error with improvement score by Friedman”

**Figure 2:**
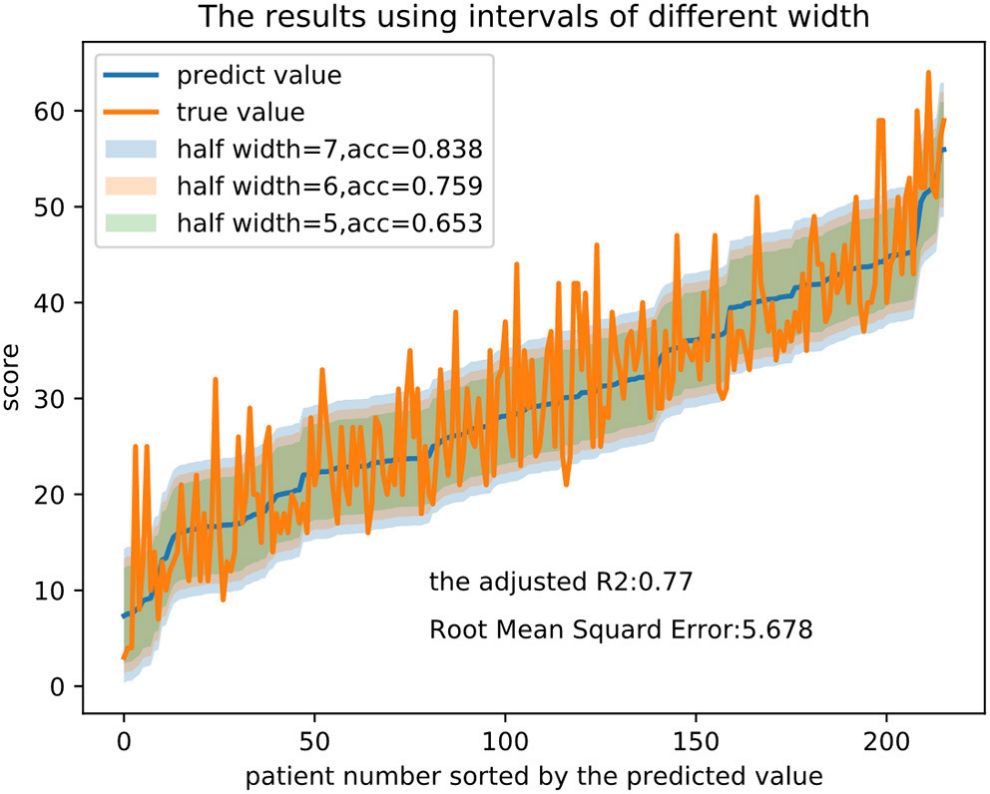
Prediction results and intervals of different widths in the RF regression model. Note that half width represents half of the width of the threshold range and acc represents the proportion of the true value that falls into the range.

Taking as an example, the random forest (RF) regression had an adjusted *R*^2^ of 0.770 and an RMSE of 5.678. The importance of each feature in the model is computed as the total reduction in the criterion introduced by that feature and is shown in Table 4. Figure 2 presents the evaluation of the predictions on the test set, with the predicted value as the midpoint of threshold ranges with half-range widths of 5, 6, and 7 for adjustment. Finally, we calculated the proportion of the true value that fell into the range as the accuracy.

### Biomarkers Influence on Parkinson's Disease Progression Model

At the end of the study, the influence of biomarkers on Parkinson's disease progression model was analyzed. First, we performed spearman correlation analysis between biomarkers and disease progression and found that the CSF amyloid and CSF α-synuclein were significantly associated with disease progression, of which the correlation coefficient were, respectively, −0.132, −0.160. The correlation analysis results of other variables are shown in Supplementary Table 1. After incorporating the two variables into feature combinations selected by feature selection based on clinical characters and scales scores and eliminating the missing data, a total of 441 cases were left. The models were reconstructed based on the feature combinations before and after the inclusion of biomarkers variables and the performance of two sets of models were compared by *t*-test. The *P*-value were 0.408 and 0.883 respectively, showing no significant difference and indicating that these biomarkers had no significant impact on the progression model within the data in the study. The *R*^2^ and RMSE of different regression models are shown in Supplementary Table 3.

## Discussion

In this study, we analyzed longitudinal data from the PPMI database to develop a predictive model for motor progression in patients with early PD. Five algorithms representing three model categories (linear, ensemble and Bayesian) were developed; and the adjusted *R*^2^ values of the models all reached 0.75. Our findings indicate that the models can practically predict the MDS-UPDRS Part III score of the coming year based on the clinically available characteristics obtained in the current year.

Our results suggest that all five algorithms (linear, ridge, Bayesian, RF and gradient boosting decision tree), categorized into linear, Bayesian and ensemble methods, have similar accuracies and features. Three common predictors were selected among the three categories: MDS-UPDRS Part III score, MOCA Score, and RBDSQ. In particular, functional status in the current year as measured by the MDS-UPDRS Part III (correlation coefficient 0.882; for more details, see the Supplementary Table 2) was consistent with and a relatively strong predictor of motor performance for the coming year. This is understandable, given that motor symptoms progress based on prior clinical features. Essentially, the results indicate that the worse one's baseline motor dysfunction is, the higher the MDS-UPDRS Part III score will be the following year.

Sleep disturbances such as disrupted circadian rhythm, insomnia, excessive daytime sleepiness (EDS), and rapid eye movement sleep behavior disorder (RBD) correlate with faster progression of motor symptoms and lower quality of life (Arnulf, 2005; Dulovic and Vos, 2018; Pagano et al., 2018). In our study, the scores of the scales used to evaluate RBD and EDS were important predictors for the progression of motor symptoms in the RF regression model, with RBD evaluation being a common predictor among the five algorithms. Subjective EDS in PD has been associated with advanced motor impairment and disease progression, male gender and the use of anti-parkinsonian medications (Arnulf, 2005; Dulovic and Vos, 2018). RBD is an aggravating factor of motor symptoms (Pagano et al., 2018), autonomic dysfunction, and dementia (Chahine et al., 2016). In a longitudinal analysis of early PD, the presence of RBD was found to predispose a patient toward a more aggressive phenotype characterized by a rapid progression of motor symptoms (Pagano et al., 2018). The promotion of neurodegeneration caused by sleep dysfunction has been proposed to drive further sleep alterations, creating a detrimental self-perpetuating cycle (Musiek and Holtzman, 2016).

Cognitive impairment at baseline is also significantly associated with faster disease progression and greater motor impairment, which has been identified in other studies (Velseboer et al., 2013; Fereshtehnejad et al., 2015; Reinoso et al., 2015). Fereshtehnejad et al. (2015) found that, besides UPDRS values, signs of cognitive impairment, orthostatic hypotension and rapid eye movement sleep behavior disorder at baseline evaluation, could suggest that patients will express a much faster decline in motor symptoms. This is mainly due to an increase in L-dopa non-responsive symptoms, which suggest a diffuse destruction of extra-nigrostriatal pathways in parallel with the nigrostriatal pathway (Velseboer et al., 2013).

There are some differences in the predictors selected among the models, which may be related to their different operating principles. The core idea underlying ensemble learning is to learn a series of basic classifiers from training data and then combine these relatively weak classifiers into a strong classifier. Then, the predictive ability of the categorical variables can be given full play, allowing the ensemble learning model to achieve a predictive effect with fewer variables. We observed this behavior among our ensemble learning models as well.

Parkinson's disease is a clinically heterogeneous disease with varied progression patterns (Qian and Huang, 2019; Beheshti et al., 2020; Haumesser et al., 2020; LeWitt et al., 2020; Shen et al., 2020). In addition to genetic factors (Gao et al., 2020), the availability of objective fluid biomarkers specifically associated with motor or cognitive trajectories of PD subtypes could allow reliable prediction of clinical outcomes (Qian and Huang, 2019; Xie et al., 2019). As for blood biomarkers, studies on blood oligomeric α-synuclein showed increased quantities in patients with PD both in serum (Williams et al., 2016) and in red blood cells(RBCs) (Wang et al., 2015; Zhao et al., 2016; Daniele et al., 2018). Similarly, plasma phosphorylated α-synuclein is higher in patients with Parkinson's disease compared with controls (Foulds et al., 2013). In addition to α-synuclein, there are several substances playing essential roles in PD. The lower plasma levels of serum superoxide dismutase (SOD), total cholesterol, high-density lipoprotein cholesterol (HDL-C), and low-density lipoprotein cholesterol (LDL-C) and increased level of high-sensitivity C-reactive protein (hsCRP) were found in PD, which might be important markers to assess the PD severity (Yang et al., 2020). One study showed a significant decrease in the ubiquitous mitochondrial creatine kinase (uMtCK) activity in the PD group and a correlation between serum uMtCK activities and the disease progression rate, duration, and age at onset in PD patients (Xu et al., 2019). Compared with healthy subjects, the serum levels of Trefoil factor 3 (TFF3) and cholinesterase activity were lower, while homocysteine (Hcy) was higher in patients with Parkinson's disease dementia (PDD) and vascular parkinsonism with dementia (VPD). Significant correlations between TFF3/ChE activity/Hcy levels and PDD/VPD severities were found, including motor dysfunction, declining cognition, and mood/gastrointestinal symptoms (Zou et al., 2018). To explore the vascular, inflammatory, metabolic risk factors of dementia in PD with type 2 diabetes mellitus (DM) (PD-DM), lower LDL, and higher fibrinogen were the most significant risk factors in PD-DM with dementia (Wang et al., 2020).

Evidence suggests that measures of CSF Aβ1-42, T-tau, P-tau181, and α-synuclein have prognostic and diagnostic potential in early-stage PD (Kang et al., 2013). Levels of α-synuclein in CSF are decreased in PD and other synucleinopathies and may serve as a marker to assist in diagnosis and prognosticating progression (Hong et al., 2010; Mollenhauer et al., 2011). Research has revealed that the NLR family pyrin domain containing 3 protein (NLRP3) inflammasome may facilitate the secretion of extracellular vesicles, as well as exosomal transmission of proteins like aggregated α-synuclein (Si et al., 2020). However, in prodromal and early PD, CSF α-synuclein does not correlate with PD's progression and does not reflect ongoing dopaminergic neurodegeneration (Mollenhauer et al., 2019). In our study, CSF biomarkers and serum uric acid had no significant impact on the progression model, indicating that easily accessible clinical assessments have sufficient capacity to predict disease progression.

Previouly, based on the PPMI database, Latourelle et al. developed comprehensive multivariable prognostic models to predict the annual rate of change in PD (Latourelle et al., 2017). There are several differences between the two studies. The first one concerns to be the methodology and objectives. The previous study mainly focused on causal analysis, which differs from our research on prediction analysis. Although the value of *R*^2^ was small in the previous study, causal analysis can also be conducted to determine the effect of independent variables on dependent variables (Latourelle et al., 2017). In Latourelle's study, the *R*^2^ value of the model was 41% for the PPMI database and 9% for the Lab-PD cohort. The shortcomings of a small *R*^2^ can be offset by a large sample size. In our work, maximization of the *R*^2^ value was critical for prediction. The adjusted *R*^2^ value for the models in each of the three categories reached 0.75, indicating that the selected features had a good predictive ability for motor scores. In general, predicting the patient's future condition can assist doctors in making decisions on when to intervene. Second, the selected features varied between the two studies. The model variables in Latourelle et al.'s study included many clinical, genetic and laboratory examinations. However, the variables in our study were all general data and clinical evaluation indicators, which could be obtained by a single doctor in the outpatient department without the need for relatively complex laboratory and imaging examinations. Thus, the use of these variables can greatly enhance the clinical practicality of our model.

Our research possesses a number of strengths. First, our prediction model was developed based on data from the previous year in order to predict annual motor symptoms, a dynamic process that has a high degree of practical clinical application value. Additionally, this study included information that was easy to obtain in the patient interview process as model features, making the model feasible for practical application. Doctors can embed the model creation process in an electronic medical record system to predict the next year's motor function automatically based on the patient's current clinical data. Second, the PPMI database contains data from different hospitals in different regions, which helps improve the accuracy of prediction.

However, there are also several limitations in our study. First, the subtypes of PD were not considered, and only uniform predictions across subtypes were made. Second, only the MDS-UPDRS Part III total score was predicted as the model result, and no subdivision prediction was made for a single item or symptom category score (such as limb rigidity, central axis slowing, tremor, gait, etc.). It is possible that the relevant trends in variability would not be reflected in the total score. Third, our study focused on the early stage of PD. Thus, the model does not apply to patients with advanced PD.

## Conclusions

In the present study, by using machine learning and routinely gathered assessments, we developed convenient predictive models synthesizing multiple clinical characteristics to provide 75% accuracy in predicting motor progression. CSF biomarkers and serum uric acid had no significant impact on the progression model, indicating that easily accessible clinical assessments have sufficient capacity to predict disease progression. The use of these models can predict motor evaluations at the individual level, allowing clinicians to tailor medical management for each patient and identify at-risk patients for future clinical trials investigating delaying motor progression. Future predictive models based on other large cohorts, assigned into training, and validation sets are needed to verify the accuracy of our results.

## Data Availability Statement

The datasets presented in this study can be found in online repositories. The names of the repository/repositories and accession number(s) can be found at: www.ppmi-info.org.

## Ethics Statement

The studies involving human participants were reviewed and approved by Parkinson's Research and the National Institute of Neurological Disorders and Stroke (1P20NS092529-01). The patients/participants provided their written informed consent to participate in this study.

## Author Contributions

L-YM and YT participated in the design of the study, drafted the manuscript, and carried out the conceptualization of the study. C-RP, Z-LC, YL, and KR performed data analysis and drafted the manuscript. TF and J-SL carried out the conceptualization of the study, reviewing, and critiquing the article at the same time. All authors contributed to the article and approved the submitted version.

## Conflict of Interest

Z-LC, YL, and KR were employed by company Gyenno Science Co. Ltd., Shenzhen, China. The remaining author declares that the research was conducted in the absence of any commercial or financial relationships that could be construed as a potential conflict of interest.
