# Supplementary material for: Motor Progression in Early-Stage Parkinson's Disease: A Clinical Prediction Model and the Role of Cerebrospinal Fluid Biomarkers
Source: Front Aging Neurosci. 2021 Jan 25;12:627199. doi: 10.3389/fnagi.2020.627199 (PMC7868416; doi:10.3389/fnagi.2020.627199)
Supplement: Supplementary file 1 [file Table_1.docx]

Table S1 Descriptive statistics of the detailed score variables of the Parkinson’s Progression Markers Initiative Study Participants

| Categorical Variables | Items | Percentages |
| --- | --- | --- |
| MDS-UPDRS Part I Cognitive Impairment | Normal | 72.363% |
|  | Slight | 23.840% |
|  | Mild | 3.376% |
|  | Moderate | 0.422% |
|  | Severe | 0.000% |
| MDS-UPDRS Part I Anxious Mood | Normal | 63.291% |
|  | Slight | 31.013% |
|  | Mild | 4.641% |
|  | Moderate | 0.844% |
|  | Severe | 0.211% |
| MDS-UPDRS Part I Apathy | Normal | 82.068% |
|  | Slight | 14.768% |
|  | Mild | 2.743% |
|  | Moderate | 0.211% |
|  | Severe | 0.211% |
| MDS-UPDRS Part I Depressed Mood | Normal | 73.840% |
|  | Slight | 21.519% |
|  | Mild | 4.219% |
|  | Moderate | 0.211% |
|  | Severe | 0.211% |
| MDS-UPDRS Part I Features of Dopamine Dysregulation Syndrome | Normal | 97.679% |
|  | Slight | 1.688% |
|  | Mild | 0.633% |
|  | Moderate | 0.000% |
|  | Severe | 0.000% |
| MDS-UPDRS Part I Fatigue | Normal | 48.312% |
|  | Slight | 38.819% |
|  | Mild | 9.072% |
|  | Moderate | 2.954% |
|  | Severe | 0.844% |
| MDS-UPDRS Part I Hallucinations and Psychosis | Normal | 96.835% |
|  | Slight | 3.165% |
|  | Mild | 0.000% |
|  | Moderate | 0.000% |
|  | Severe | 0.000% |
| Any QUIP disorder | No QUIP Disorders | 77.215% |
|  | Any 1 or more disorders | 22.785% |
| QUIP disorder - Buying | No Buying disorder | 96.624% |
|  | Buying disorder | 3.376% |
| QUIP disorder - Eating | No Eating disorder | 89.662% |
|  | Eating disorder | 10.388% |
| QUIP disorder - Gambling | No Gambling disorder | 99.156% |
|  | Gambling disorder | 0.844% |
| QUIP disorder - Hobbies | No Hobbies disorder | 91.983% |
|  | Hobbies disorder | 8.017% |
| QUIP disorder - Punding | No Punding disorder | 94.093% |
|  | Punding disorder | 5.907% |
| QUIP disorder - Sex | No Sex disorder | 97.257% |
|  | Sex disorder | 2.743% |
| QUIP disorder - Walking or Driving | No Walking/Driving disorder | 98.734% |
|  | Walking/Driving disorder | 1.266% |

Table S2 The correlation coefficient and probability value between the remaining variables and the outcome event

| Variables | Correlation coefficient | Probability value | Variables | Correlation coefficient | Probability value |
| --- | --- | --- | --- | --- | --- |
| MDS-UPDRS Part Ⅲ Score | 0.882 | 0.000 | ESS Score | 0.104 | 0.002 |
| Duration (Months) | 0.269 | 0.000 | Initial symptom (at diagnosis) - Postural Instability | 0.079 | 0.022 |
| MDS-UPDRS Part I Apathy | 0.197 | 0.000 | Family History | 0.069 | 0.044 |
| SCOPA-AUT Total Score | 0.196 | 0.000 | MDS-UPDRS Part I Hallucinations and Psychosis | 0.056 | 0.101 |
| MDS-UPDRS Part I Fatigue | 0.154 | 0.038 | Initial symptom (at diagnosis) - Resting Tremor | 0.032 | 0.353 |
| MDS-UPDRS Part I Depressed Mood | 0.142 | 0.000 | MDS-UPDRS Part I Features of Dopamine Dysregulation Syndrome | 0.022 | 0.523 |
| Age at symptom onset | 0.138 | 0.000 | TD/PIGD classification (OFF) | 0.022 | 0.525 |
| GDS Score | 0.132 | 0.000 | Initial symptom (at diagnosis) - Bradykinesia | 0.019 | 0.573 |
| MDS-UPDRS Part I Anxious Mood | 0.131 | 0.000 | QUIP Score | -0.021 | 0.549 |
| RBDSQ Score | 0.126 | 0.000 | Side most affected at PD onset | -0.061 | 0.075 |
| MDS-UPDRS Part I Cognitive Impairment | 0.125 | 0.000 | Gender | -0.072 | 0.035 |
| Initial symptom (at diagnosis) - Rigidity | 0.109 | 0.001 | MOCA Score | -0.221 | 0.000 |
| CSF amyloid | -0.132 | 0.001 | CSF α-synuclein | -0.160 | 0.000 |
| CSF amyloid/CSF α-synuclein | -0.020 | 0.594 | Serum Uric Acid | 0.006 | 0.876 |

MDS-UPDRS = Movement Disorder Society-Unified Parkinson’s Disease Rating Scale

SCOPA = Scales for Outcomes in Parkinson’s disease

ESS = Epworth Sleepiness Scale

RBDSQ = REM Sleep Behavior Disorder Screening Questionnaire

TD = Tremor Dominant

PIGD = Postural Instability and Gait Difficulty

GDS = Geriatric Depression Scale

QUIP = Questionnaire for Impulsive-Compulsive Disorders in Parkinson’s Disease

MOCA = Montreal Cognitive Assessment

CSF = Cerebrospinal Fluid

Table S3 The R2 and RMSE of different regression models before and after adding CSF biomarkers

| Regression models | Linear regression | Ridge regression | Bayesian regression | Random forest regression | Gradient boosting regression |
| --- | --- | --- | --- | --- | --- |
| R2 (before adding CSF biomarkers) | 0.743 | 0.743 | 0.737 | 0.721 | 0.722 |
| RMSE (before adding CSF biomarkers) | 5.673 | 5.678 | 5.718 | 5.883 | 5.931 |
| R2 (after adding CSF biomarkers) | 0.739 | 0.738 | 0.731 | 0.713 | 0.711 |
| RMSE (after adding CSF biomarkers) | 5.666 | 5.671 | 5.718 | 5.900 | 5.993 |
